# Serum circular RNA hsa_circ_0000702 as a novel biomarker for diagnosis of gastric cancer

**DOI:** 10.1002/jcla.24842

**Published:** 2023-01-16

**Authors:** Wentao Yuan, Ronghua Fang, Chunyan Mao, Hongmei Chen, Bojun Tai, Hui Cong

**Affiliations:** ^1^ Department of Laboratory Medicine Affiliated Hospital of Nantong University Nantong China; ^2^ Medical School of Nantong University Nantong China; ^3^ Vip Ward, Affiliated Hospital of Nantong University Nantong China; ^4^ Department of Infectious Diseases Affiliated Hospital of Nantong University Nantong China; ^5^ Department of Blood Transfusion Affiliated Hospital of Nantong University Nantong China

**Keywords:** biomarkers, circular RNA, gastric cancer, hsa_circ_0000702, serum

## Abstract

**Background:**

There is mounting evidence that Circular RNAs (circRNAs) are essential for the initiation and development of gastric cancer (GC). In this study, we further investigated the clinical importance and applicability of serum hsa_circ_0000702 in the diagnosis and treatment of GC.

**Methods:**

Sanger sequencing, agarose gel electrophoresis, and RNase R assay were used to confirm the origin, alterations, and stability of hsa_circ_0000702 in GC patients. Real‐time quantitative reverse transcription‐polymerase chain reaction (qRT‐PCR) was used to detect the expression level of hsa_circ_0000702 in GC cell lines, serum, and tissues. Additionally, receiver operating characteristic (ROC) curves were built to evaluate their prognostic value and how well they would work in conjunction with popular biochemical markers for GC. Finally, real‐time dynamic monitoring was used to assess its prognostic usefulness.

**Results:**

Hsa_circ_0000702 exhibited the fundamental traits of circRNA. Hsa_circ_0000702 had good sensitivity, specificity, and stability. It was discovered that hsa_circ_0000702 was down‐regulated in GC cell lines, serum, and tissues, and that the level of tumor differentiation and tumor node metastasis (TNM) staging were connected with serum hsa_circ_0000702. The area under the ROC curve of serum hsa_circ_0000702 was calculated to be 0.745 (95% CI: 0.669–0.821), indicating high diagnostic efficacy. The diagnostic value was greatly increased by combining serum CEA and CA19‐9. Finally, preoperative and postoperative dynamic monitoring revealed serum hsa_circ_0000702 to be of clinical application.

**Conclusion:**

Serum hsa_circ_0000702 was variably expressed in GC patients, indicating that serum hsa_circ_0000702 may be a novel biomarker for GC diagnosis and dynamic monitoring.

## Graphical abstract

Sanger sequencing, agarose gel electrophoresis, and RNase R assay were used to confirm the origin, alterations, and stability of the hsa_circ_0000702 in GC patients. Real‐time quantitative polymerase chain reaction (qRT‐PCR) was used to detect the expression level of hsa_circ_0000702 in gastric cancer cell lines, serum, and tissues. Additionally, receiver operating characteristic (ROC) curves were built to evaluate their prognostic value and how well they would work in conjunction with popular biochemical markers for GC. Finally, real‐time dynamic monitoring was used to assess its prognostic usefulness.

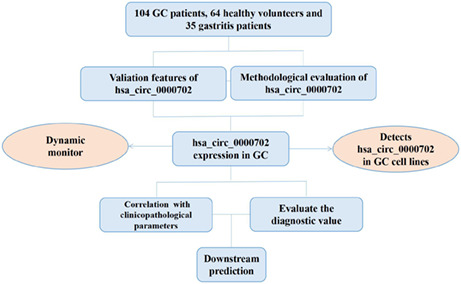

## 1 INTRODUCTION

One of the most prevalent major malignant tumors in the world, gastric cancer (GC) continues to have a high incidence and fatality rate. GC is the third most common malignant tumor in China in terms of incidence and fatality rate, which poses a severe threat to human life and health. According to the most recent statistics, GC incidence and fatality rates are rising as people age.^1^, ^2^ Even though surgery and adjuvant therapy have improved the prognosis for GC patients recently, the disease still has a dismal survival rate because most patients present with it have subtle early‐stage symptoms and are already in the middle or late stages when they are identified. Currently, biomarkers including CEA, CA19‐9, and CA72‐4 are frequently utilized in the detection of patients with GC, although their diagnosis is somewhat limited due to their low sensitivity or specificity.^3^ Additionally, PG I, PG II, and PG I/II have less usefulness in the diagnosis of GC.^4^ Finding new biomarkers for the early diagnosis, therapy, and prognostic evaluation of GC becomes critical and necessary.

Circular RNA (circRNA) is a novel type of RNA molecule that can be broadly expressed in a variety of cells and tissues and is distinguished by a covalently closed loop structure lacking a 5′ end cap and a 3′ end poly (A) tail. CircRNAs are more stable and more difficult to degrade than mRNA, microRNA (miRNA), and long non‐coding RNA (lncRNA). CircRNAs are closely related to the occurrence and development of tumors. Many studies have shown that circRNAs can be used in the diagnosis and treatment of tumors. For instance, circ‐ZEB1 promotes PIK3CA expression and affects the proliferation and apoptosis of hepatocellular carcinoma cells by silencing miR‐199a‐3p,^5^ hsa_circ_0006089 promotes TGFB1 expression by sponging miR‐361‐3p, which facilitates the growth, metastasis, glycolysis, and angiogenesis of gastric cancer cells.^6^ Additionally, circRNA has the potential to be useful as a biomarker because it is extensively dispersed and may be found in bodily fluids like blood, saliva, and urine. In the serum of patients with GC, for instance, hsa_circ_0003195 and hsa_circ_0035445 were down‐regulated with an area under curve (AUC) as high as 85%.^7^, ^8^ These new biomarkers offer improved sensitivity and specificity when compared to CEA and CA19‐9. Therefore, circRNA is anticipated to be a potential biomarker for early cancer detection as well as cancer diagnosis and prognosis.

In the current study, database searching revealed that hsa_circ_0000702 was dysregulated in GC. There were, however, no pertinent findings on the degree of expression and clinical importance of hsa_circ_0000702 in GC. In order to investigate the viability of hsa_circ_0000702 as a biomarker for the diagnosis and treatment of GC, we concentrated on examining the expression levels of hsa_circ_0000702 in GC cell lines, serum, and tissues as well as its association with clinical indices of GC patients.

## 2 MATERIALS AND METHODS

### 2.1 Patient selection and sample collection

Randomly chosen participants for this study included patients who visited Nantong University Hospital between August 2021 and August 2022, as well as healthy volunteers who had a physical examination. A total of 17 pairs of GC tissues and their corresponding adjacent tissues, 104 peripheral serum samples from patients with GC (all of whom were pathologically confirmed to have the disease and were all discovered without having received any prior treatment), and 23 peripheral serum samples from corresponding post‐operative GC patients. Additionally, peripheral serum samples were taken from 64 healthy people who were examined during this period as well as 35 patients with gastritis who had the condition. For subsequent processing, all samples were kept in −80°C refrigerator. The research protocol was approved by the medical ethics committee of Nantong University Affiliated Hospital (Approval No. 2021‐L033).

### 2.2 Cell culture

Human GC cell lines (MKN‐45, SGC‐7901, MGC‐803, and AGS) and human gastric epithelial cells (GES‐1) were purchased from the Stem Cell Bank of the Chinese Academy of Sciences, where GES‐1 was used as normal control. RPMI‐1640 medium (Corning), 10% fetal bovine serum (FBS, Gibco), and 1% double antibodies (penicillin and streptomycin) were prepared and placed in an overnight culture in a humidified incubator at 37°C containing 5% CO_2_.

### 2.3 RNA extraction, reverse transcription (RT), and real‐time quantitative reverse transcription‐polymerase chain reaction (qRT‐PCR)

Total serum RNA was extracted using a sorbent column method according to the manufacturer's instructions by adding 900 μl of lysate RLS per 300 μl of serum using a serum RNA extraction kit (BioTeke). A certain amount of total RNA was taken according to the concentration of RNA and reverse transcribed according to the instructions on the kit. The relative expression of serum circRNA was measured by qRT‐PCR. The primer sequences were as follows. 18 S rRNA (F: 5′‐GTAACCCGTTGAACCCCATT‐3′; R: 5′‐CCATCCAATCGGTAGTAGCG‐3′), hsa_circ_0000702 (F: 5′‐GATGGAGTTGATGCAAGGCC‐3′; R: 5′‐GCTCGGTCAAACATCTCTCTCTC‐3′).

### 2.4 Extraction of gDNA and RNase R assays

#### 2.4.1 gDNA extraction

Cellular gDNA was extracted according to the operating instructions provided in the gDNA extraction kit. After that, subsequent qRT‐PCR experiments were performed. RNase R Assays: Take two 5 μg of cellular RNA and put them in 200 μl of RNase‐free EP tubes. One was treated with 3 U RNase R and the other was left untreated to make a 20 μl reaction system and incubated for 15 min at 37°C. The one treated with RNase R was then put into 70°C for 10 min to inactivate the RNase in the tube. The two RNAs were then reverse‐transcribed into cDNA for subsequent RT‐qPCR experiments.

### 2.5 Agarose gel electrophoresis

A gel was made by adding 1.5 g of agarose to 50 ml of 1 × Tris Acidate‐EDTA (TAE) solution, microwave heating until dissolved, and then adding 2 μl of ethidium bromide after cooling. Place the solidified gel in the electrophoresis tank, mix 5 μl of PCR product and 1 μl of buffer, respectively, and add to the sample wells. Electrophoresis parameters were set: voltage (V) ‐ 110; amperage (A) ‐ 210; time was about 40 min, and the results were observed by the gel imaging system.

### 2.6 Nucleoplasmic separation assay

Nucleoplasmic separation of gastric cancer cell lines was performed by the Nucleoplasmic and Cytoplasmic Protein Extraction Kit (Beyoncé). Cells were collected and rinsed with PBS before handling on ice. Cell Plasma Protein Extraction Reagent A was added and the cells were suspended and dispersed by vigorous shaking for 10 min in an ice bath. Afterwards, Cell Plasma Protein Extraction Reagent B is added and the supernatant is extracted by centrifugation for 5 min. After that, nucleoprotein extraction reagent was added, and the cells were shaken back and forth for 30 min in an ice bath, and the supernatant was extracted as nucleoprotein after centrifugation.

### 2.7 Statistical analysis

Statistical analyses of clinicopathological parameters were performed using SPSS 27.0 (IBM 226 Corporation), and graphs were drawn by GraphPad Prism 9.0 (GraphPad Software Inc.). Statistical analyses were performed using one‐way ANOVA and independent samples *t*‐test. The clinical diagnostic effect of hsa_circ_0000702, CEA, and CA19‐9 was evaluated by the AUC area under the ROC curve. *p* < 0.05 was considered statistically significant.

## 3 RESULTS

### 3.1 Structure verification of hsa_circ_0000702

The GEO database search found that the expression of hsa_circ_0000702 in GC tissues was abnormal. The mature transcript length of hsa_circ_0000702 was 1106 bp, and it was obtained from the parent gene CHD9, which is located at chr16: 53288349–53,308,214, according to the human circRNA database ‐ Circbank (http://www.circbank.cn/index.html).

**FIGURE 1:**
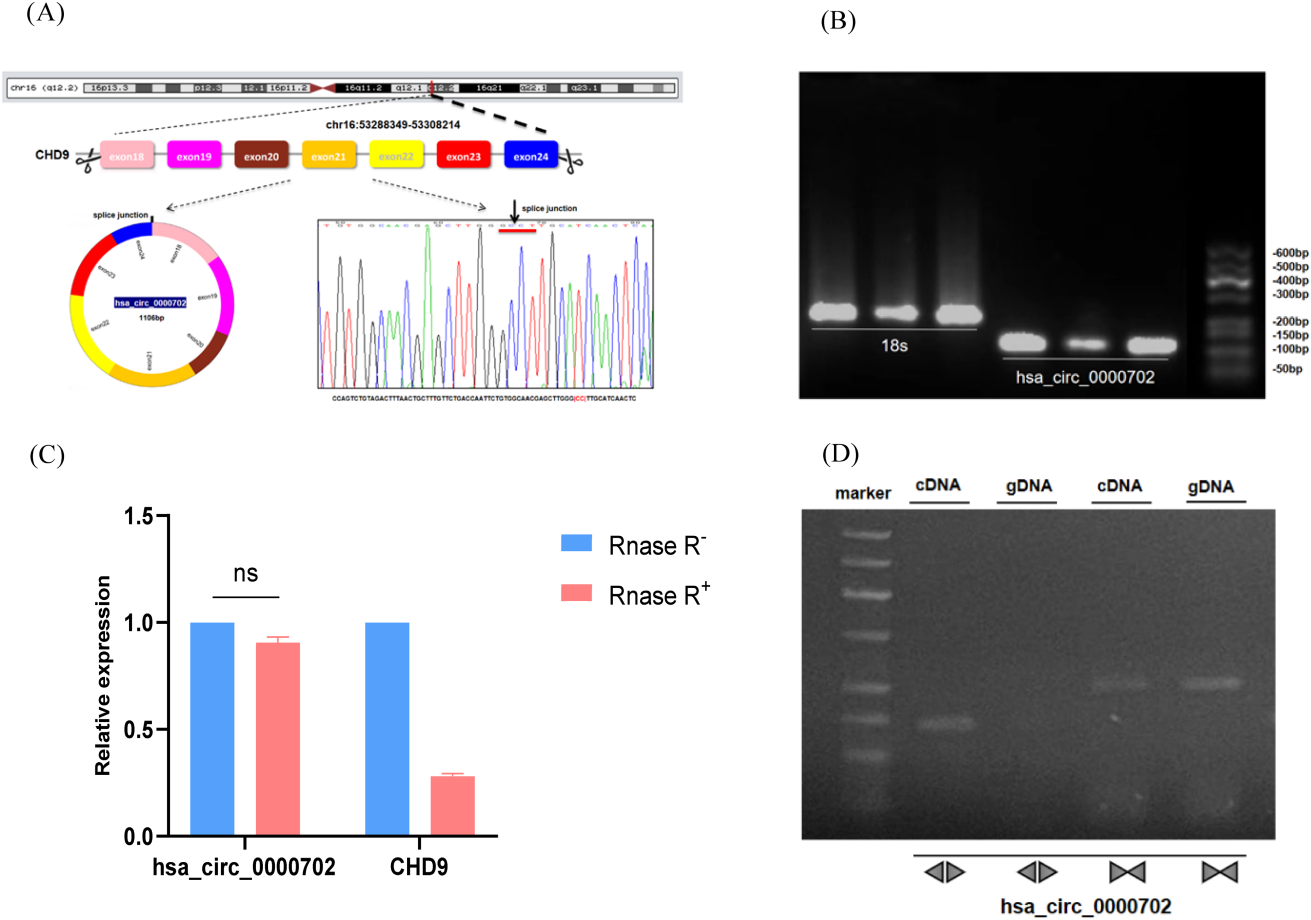
Features of hsa_circ_0000702. (A) Location and origin of hsa_circ_0000702 and Sanger sequencing to verify the cyclization site. (B) hsa_circ_0000702 Verification of primer length (115 bp) by agarose gel electrophoresis. (C) Stability of hsa_circ_0000702 confirmed by RNase R assay. (D) Verifying the ring structure of hsa_circ_0000702

The CircPrimer software analyzed that hsa_circ_0000702 consisted of exons 18, 19, 20, 21, 22, 23, and 24. The expression level of hsa_circ_0000702 was detected by primer design, and its product was detected by agarose gel electrophoresis. The electrophoretic band was 115 bp, which was consistent with the size of the primer amplification product. The reverse splice site of hsa_circ_0000702 was further identified using Sanger sequencing (Figure 1A,B).

Considering that circRNAs usually form closed loops by reverse splicing, a Divergent primer that can reverse amplify circular molecules and a Convergent primer that can amplify both linear and circular molecules were designed based on the hsa_circ_0000702 cyclization site, and the total RNA was treated with RNase R. qRT‐PCR revealed that the expression level of CHD9 mRNA was significantly decreased after RNase R assay, while that of hsa_circ_0000702 was only slightly decreased. In addition, qRT‐PCR was performed using genomic DNA (gDNA) and cDNA as templates. Agarose electrophoresis results showed that PCR products using cDNA as a template could amplify hsa_circ_0000702, while the control group using gDNA as a template showed negative results (Figure 1C,D). The above experiments confirmed that hsa_circ_0000702 is a structurally stable closed‐loop RNA.

### 3.2 Methodological evaluation of serum hsa_circ_0000702

**FIGURE 2:**
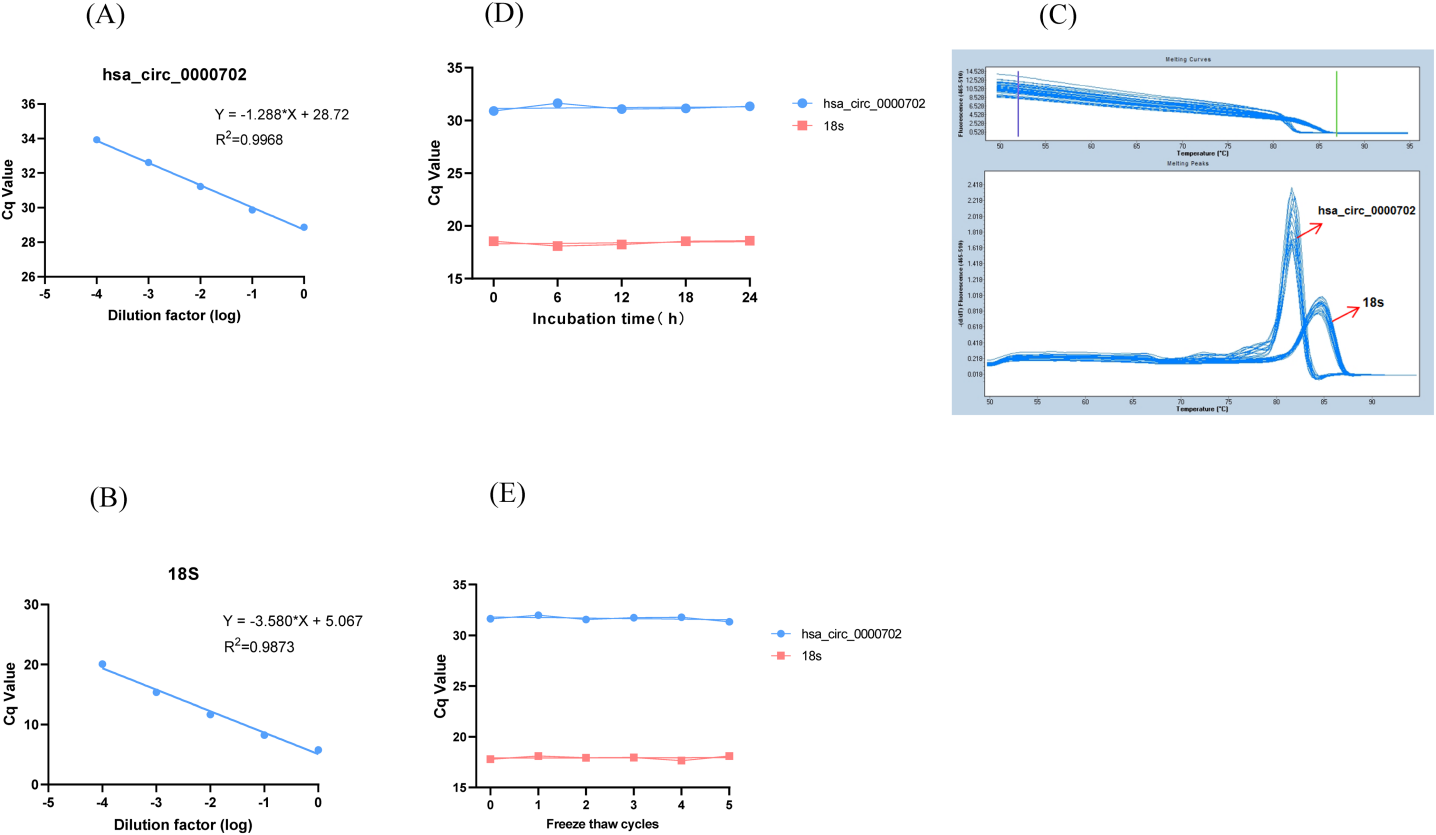
Methodological evaluation of hsa_circ_0000702. (A, B) Standard curves of hsa_circ_0000702 (*R* ^2^ = 0.9968) and 18s (*R* ^2^ = 0.9873). (C) PCR single peak curves of hsa_circ_0000702 and 18s. (D, E) Stability of hsa_circ_0000702 and 18s during incubation at room temperature and repeated freeze–thaw

In order to better verify the stability of hsa_circ_0000702, the reverse transcription product cDNA was diluted at several concentrations such as 1:10, 1:100, 1:1000, and 1:10 000, the changes in Cq values were compared for linearity. The results showed that the *R* ^2^ of hsa_circ_0000702 was 0.9968, and the regression equation was *Y* = −1.288 * *X* + 28.72. Meanwhile, the *R* ^2^ of the selected internal reference (18S rRNA) was 0.9873, and the regression equation was *Y* = −3.580 * *X* + 5.067. It indicated that the two had a good linear correlation and met the experimental requirements (Figure 2A,B). Additionally, 20 serum samples (including GC patients and healthy negative controls) were randomly selected, mixed, and divided into 20 aliquots. Total RNA was extracted from 10 samples of the same batch. Intra‐batch coefficient of variation (CV) was calculated based on Cq values. The remaining 10 samples were averaged to take 1 sample per day and the corresponding inter‐batch CV values were calculated. The results showed that the inter‐batch and intra‐batch CV values were less than 5%, indicating good precision of serum hsa_circ_0000702 (Table 1). Given the significance of the stability in liquid biopsies, we extracted and detected the expression of hsa_circ_0000702 in the serum by incubating them at room temperature for 0, 6, 12, 18, and 24 h. We also extracted and detected the expression of hsa_circ_0000702 by repeatedly freezing and thawing the sera for 0, 1, 2, 3, 4, and 5. Results from qRT‐PCR revealed that serum hsa_circ_0000702 expression variations were not statistically different (all *p* > 0.05), and their melting curves were all single peaks with high stability and specificity (Figure 2C–E). In conclusion, qRT‐PCR has an excellent performance in all areas for detecting serum hsa_circ_0000702.

**TABLE 1:** The intra‐assay and inter‐assay repeatability difference of hsa_circ_0000702

	hsa_circ_0000702	18s
Intra‐assay		
Mean ± SD	31.260 ± 0.100	18.591 ± 0.031
CV (%)	0.32	0.16
Inter‐assay		
Mean ± SD	30.943 ± 0.286	17.585 ± 0.146
CV (%)	0.92	0.85

### 3.3 Verification of hsa_circ_0000702 low expression in GC

**FIGURE 3:**
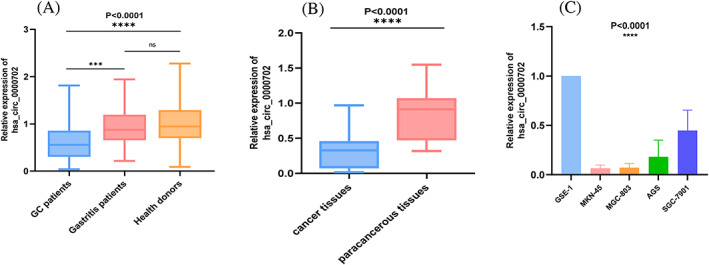
hsa_circ_0000702 low expression in GC. (A) Detection of serum expression levels in hsa_circ_0000702 in GC patients (*n* = 104), healthy volunteers (*n* = 64) and gastritis patients (*n* = 35). Indicated statistical significance. (B) Detection of expression levels in hsa_circ_0000702 in 17 pairs of GC tissues and corresponding adjacent tissue samples. Indicated statistical significance. (C) Detects the expression levels of hsa_circ_0000702 in four GC cell lines. Indicated statistical significance. ****p < 0.0001, ns indicates *p* > 0.05

qRT‐PCR was used to evaluate the expression levels of hsa_circ_0000702 in the serum of 104 patients with GC, 35 patients with benign gastritis, and 64 healthy controls. The results showed that the expression levels of hsa_circ_0000702 in the serum of GC patients were significantly lower than those of both benign gastritis patients and healthy controls (*p* = 0.0004, *p* < 0.0001), whereas the difference in serum hsa_circ_0000702 levels between benign gastritis patients and healthy controls was not statistically significant (*p* = 0.2379) (Figure 3A). In addition, the expression level of hsa_circ_0000702 was detected in 17 pairs of GC tissues and corresponding adjacent tissue samples, and the results confirmed that it was also significantly down‐regulated in GC tissues (Figure 3B). After that, the expression levels of hsa_circ_0000702 in GC cells (MKN‐45, SGC‐7901, MGC‐803, and AGS) and human gastric epithelial cells (GES‐1) were detected, and the results showed that the expression in cells was consistent with the expression in serum and tissues. Among them, the most significant difference in MKN‐45 expression was observed (Figure 3C).

### 3.4 Correlation of serum hsa_circ_0000702 with clinicopathological features in GC patients

To determine whether serum hsa_circ_0000702 levels correlated with the clinicopathological characteristics of GC, GC patients were grouped according to clinical case parameters such as age, gender, tumor size, degree of differentiation, whether neuro/vascular differentiation, tumor node metastasis (TNM), and Lauren typing. Based on the median serum hsa_circ_0000702 level, 104 GC patients were subdivided into two groups (high group, *n* = 52, and low group, *n* = 52). The results revealed that the expression level of serum hsa_circ_0000702 was associated with the degree of tumor differentiation (*p* = 0.024) and TNM stage (*p* = 0.003). However, there was no significant correlation with other clinicopathological characteristics such as age, gender, tumor size, whether neurovascular differentiation, and Lauren's staging (*p* > 0.05) (Table 2).

**TABLE 2:** Correlation of hsa_circ_0000702 expression levels with clinicopathological parameters in GC patients

Characteristic	*n*	hsa_circ_0000702		*p* value
		High	Low	
Total	104	52	52	
Gender				
Male	73	38	35	0.334
Female	31	14	17	
Age (years)				
<70	47	22	25	0.347
≥70	57	30	27	
Tumor size (cm)				
<4	61	31	30	0.500
≥4	43	21	22	
Histological differentiation				
Well‐moderate	59	24	35	0.024*
Poor	45	28	17	
Lymphatic metastasis (yes/no)				
Yes	59	27	32	0.214
No	45	25	20	
Neural/vascular differentiation (yes/no)				
Yes	64	29	35	0.157
No	40	23	17	
TNM stage				
I and II	51	33	18	0.003**
III and IV	53	19	34	
Lauren				
Intestinal type	36	20	16	0.528
Diffuse type	31	13	18	
Mixed type	37	19	18	
CEA				
Negative	85	40	45	0.155
Positive	19	12	7	
CA19‐9				
Negative	52	39	42	0.319
Positive	52	12	10	

*Note*: Statistical analyses were carried out using Pearson *χ* ^2^ test. *p < 0.05, **p < 0.01 was considered significant.

### 3.5 Serum hsa_circ_0000702 has the potential as a diagnostic GC marker

**FIGURE 4:**
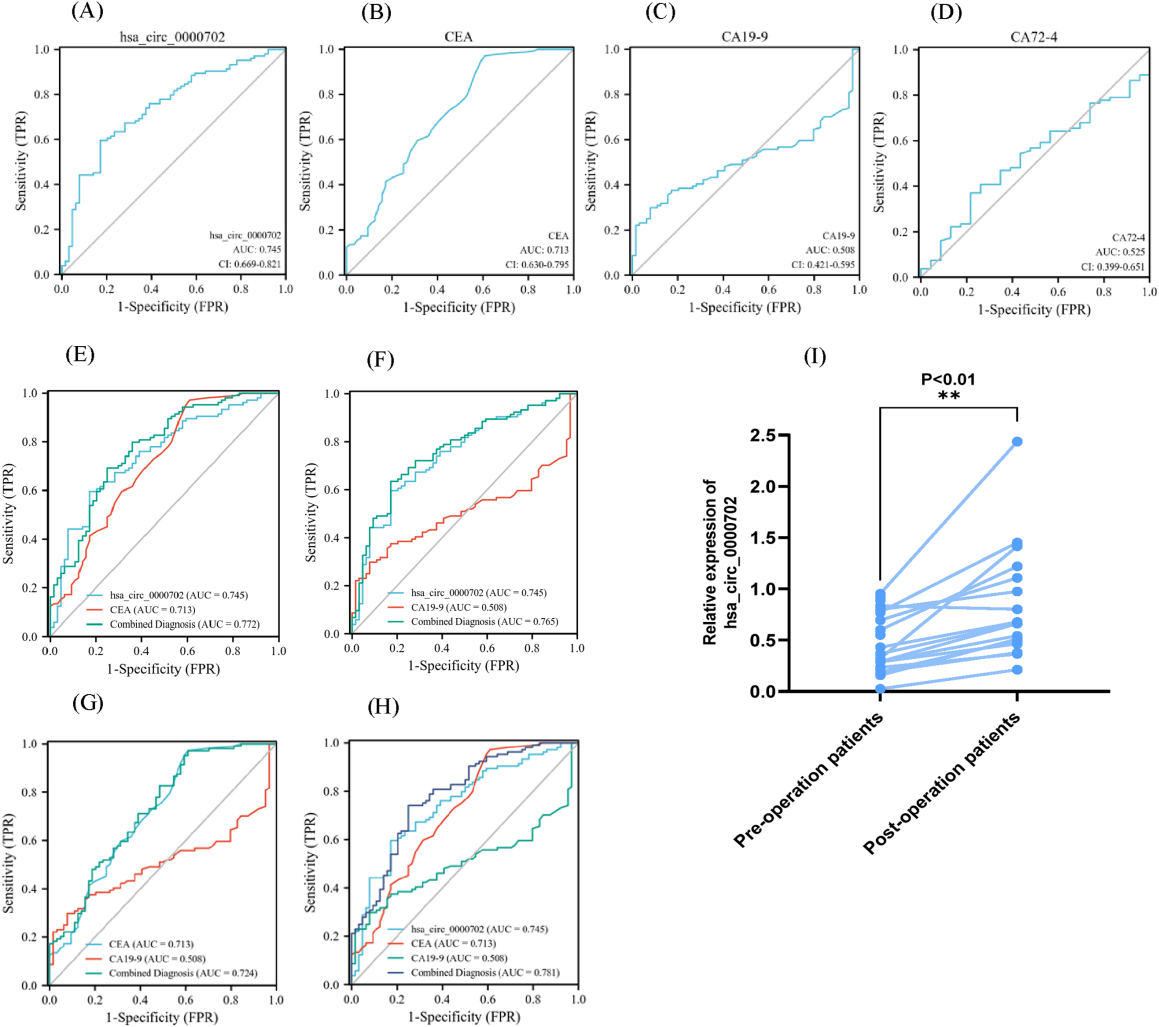
Evaluation of the diagnostic value of hsa_circ_0000702 in GC. (A–C) ROC analysis using serum hsa_circ_0000702, CEA, and CA19‐9 independently to differentiate GC patients (*n* = 104) from healthy controls (*n* = 64). (D) ROC analysis using serum CA72‐4 independently to differentiate GC patients (*n* = 81) from healthy controls (*n* = 23). (E–H) ROC analysis with separate joint diagnostic distinctions between GC patients (*n* = 104) and healthy controls (*n* = 64). (I) Changes in hsa_circ_0000702 expression before and after treatment (**p < 0.01)

To test the diagnostic efficacy of serum hsa_circ_0000702 in GC, we performed ROC curve analysis of hsa_circ_0000702, CEA, and CA19‐9 in 104 GC patients and 64 healthy controls. The results showed that the area under curve (AUC) value of hsa_circ_0000702 was 0.745 (95% CI = 0.669–0.821), and its AUC was higher than that of CEA (0.713, 95% CI = 0.630–0.795) and CA19‐9 (0.508, 95% CI = 0.421–0.595). In addition, an additional ROC curve analysis of CA72‐4 was performed for selected GC patients and healthy controls. The combined ROC analysis revealed an AUC value of 0.772 (95% CI = 0.698–0.845) for hsa_circ_0000702 after combined analysis with CEA, and 0.765 (95% CI = 0.644–0.805) after combined analysis with CA19‐9, while the AUC value after the combination of all three was 0.781 (95% CI = 0.698–0.845), which were higher than those of any two biomarkers alone or in combination, and their combined diagnostic sensitivity was also as high as 84.62% (Table 3). The results showed that the AUC value of CA72‐4 was 0.525 (95% CI = 0.399–0.651) (Figure 4A–H). The above analysis indicates that hsa_circ_0000702 has good diagnostic efficacy and that the combination of biomarkers can compensate for the limitations of a single biomarker or any two biomarkers.

**TABLE 3:** Evaluation of the diagnostic value of hsa_circ_0000702, CEA, CA199, and combinations for GC patients and healthy controls

	SEN	SPE	ACCU	PPV	NPV	AUC (95% CI)
CEA	17.31% (18/104)	93.75% (60/64)	46.43% (78/168)	81.82% (18/22)	41.10% (60/146)	0.713 (0.630–0.795)
CA19‐9	22.12% (23/104)	95.31% (61/64)	50.00% (84/168)	88.46% (23/26)	42.96% (61/142)	0.508 (0.421–0.595)
hsa_circ_0000702	82.69% (86/104)	48.44% (31/64)	69.64% (117/168)	72.27% (86/119)	63.27% (31/49)	0.745 (0.669–0.821)
CEA + CA19‐9	32.69% (34/104)	89.06% (57/64)	54.17% (91/168)	82.93% (34/41)	44.88% (57/127)	0.724 (0.644–0.805)
hsa_circ_0000702 + CEA	83.65% (87/104)	46.88% (30/64)	69.64% (117/168)	71.90% (87/121)	63.83% (30/47)	0.772 (0.698–0.845)
hsa_circ_0000702 + CA19‐9	83.65% (87/104)	46.88% (30/64)	69.64% (117/168)	71.90% (87/121)	63.83% (30/47)	0.765 (0.644–0.805)
hsa_circ_0000702 + CEA + CA19‐9	84.62% (88/104)	43.75% (28/64)	69.05% (116/168)	70.97% (88/124)	63.63% (28/44)	0.781 (0.698–0.845)

To determine whether hsa_circ_0000702 could be a key prognostic molecule, we compared the preoperative and postoperative serum hsa_circ_0000702 expression levels in 23 GC patients and found that the expression of serum hsa_circ_0000702 was significantly higher in patients 1 week after surgery compared with the initial diagnosis (*p* = 0.0091) (Figure 4I). This shows that for the dynamic monitoring of GC patients following surgical treatment, hsa_circ_0000702 may have some clinical application value.

### 3.6 Downstream prediction of hsa_circ_0000702 in GC

**FIGURE 5:**
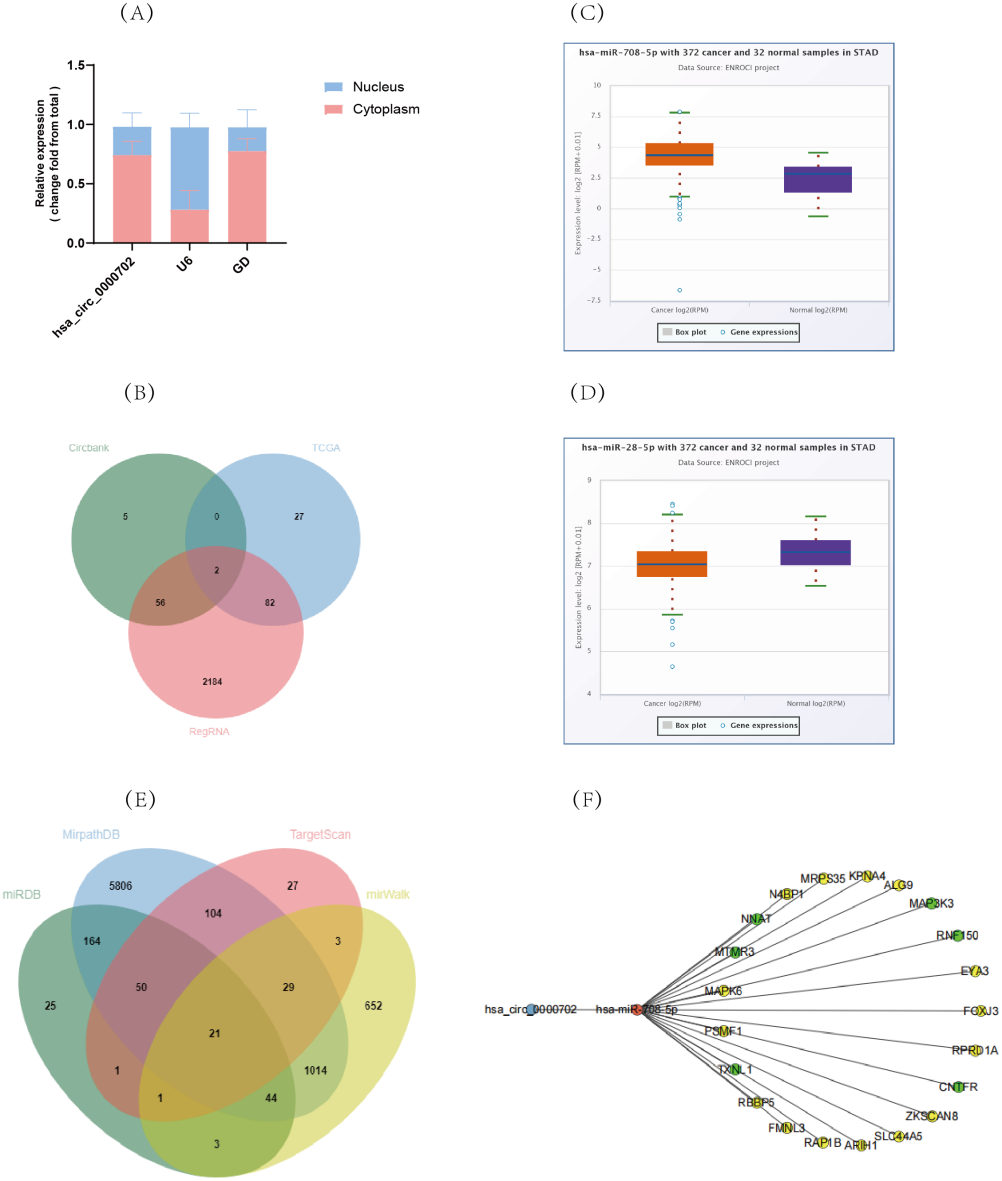
hsa_circ_0000702 downstream prediction in GC. (A) Localization of hsa_circ_0000702 in GC cells determined by nucleoplasmic separation assay. (B) Predicted target miRNA of hsa_circ_0000702 by the CircBank, RegRNA, and TCGA databases. (C, D) Differential expression of miRNAs in gastric cancer predicted by the starbase database. (E) Prediction of miRNA target mRNAs by the miRDB, MirPathDB, mirWalk, and TargetScan databases. (F) hsa_circ_0000702‐miRNA‐mRNA regulatory network. According to the starbase database, the molecules with elevated expression in stomach cancer are shown in yellow, whereas those with decreased expression are shown in green

To further investigate the mechanism of hsa_circ_0000702 action in GC cells. We showed, by nucleoplasmic separation assay, that hsa_circ_0000702 was slightly higher in the cytoplasm than in the nucleus (Figure 5A), indicating that it may play a regulatory role through competing endogenous RNA mechanisms. Therefore, the differentially expressed miRNAs in gastric cancer were first analyzed by RNA‐seq data from the TCGA database. 111 differentially expressed miRNAs were identified by R language analysis (*p* < 0.05, |logFC| ≥ 1). Then, we predicted downstream miRNAs that could be sponged by hsa_circ_0000702 by searching bioinformatics databases (CircBank and RegRNA) and performed Venn analysis of these target miRNAs with differential miRNAs from TCGA database, and a total of 2 miRNAs were screened (hsa‐miR‐708‐5p, hsa‐miR‐28‐5p) (Figure 5B). The differential expression of these two miRNAs in gastric cancer was predicted and analyzed by the Starbase database (Figure 5C,D). Among them, hsa‐miR‐708‐5p expression was up‐regulated, indicating that hsa‐miR‐708‐5p might be involved in the binding of hsa_circ_0000702 and thus regulating gastric carcinogenesis and progression. After that, the possible downstream mRNAs of hsa‐miR‐708‐5p were predicted and analyzed by the miRDB, MirPathDB, mirWalk, and TargetScan databases, and a total of 21 target mRNAs were screened by Venn analysis (Figure 5E). The circRNA‐miRNA‐mRNA axis was plotted using Cytoscape software (Figure 5F), which provided a new direction for the subsequent study of hsa_circ_0000702.

## 4 DISCUSSION

Gastric cancer used to be a cancer with high incidence in China. Although the incidence of lung cancer has soared, the incidence of GC is still close behind. However, according to the World Health Organization (WHO): with 480,000 new cases and 370,000 deaths per year, China is still a high‐incidence area for GC worldwide, and GC is not far away.^9^, ^10^ Gastroscopy is currently the preferred modality to confirm the diagnosis of GC. Under gastroscopy, most gastric mucosal lesions and early GC can be detected. However, it is less suitable for screening because it is more invasive and difficult for patients to accept. And common GC tumor markers such as CEA and CA19‐9 can generally be used for screening, but they lack certain sensitivity and specificity, and cannot diagnose gastric lesions in the first place. The gastric function index tests such as serum pepsinogen (PG), gastrin‐17 (G‐17), and Helicobacter pylori (Hp) are also only used to determine whether the gastric mucosa is damaged and whether the patient's digestive function is affected. This requires us to develop new more non‐invasive or minimally invasive tests with high sensitivity to improve the efficacy of early screening and prognostic treatment of cancer. And today, globally, the cancer burden is expected to increase by 50% by 2040 due to the increasing aging of the population.^11^ For China, incorporating cancer prevention and treatment interventions into health plans will help to reduce the future cancer burden and promote the development of innovative anti‐cancer drugs with a two‐pronged approach of ‘prevention + treatment’ to better reduce the cancer burden and protect people's health.^12^ Therefore, the search for novel noninvasive biomarkers for GC screening has become a top priority.

Circular RNAs (circRNAs) have a covalently closed circular conformation. Unlike traditional linear RNAs (containing 5′ and 3′ ends), circRNA molecules are not affected by RNA exonucleases, are more stably expressed, and are less susceptible to degradation. circRNAs can be classified into four types, namely exon circRNAs, intronic circRNAs, and exon‐Different types of circRNAs are distributed in different locations and play different roles. CircRNAs were previously classified as non‐coding RNAs due to their highly conserved structure. However, recent research has shown that circRNAs have the potential to encode proteins.^13^ CircRNAs initiates protein translation via the internal ribosome entry sites (IRESs), the N^6^‐methyladenosine (m^6^A) modification, and rolling circle amplification, whose protein products can induce the formation of diseases such as tumors.^14^, ^15^ For example, SMO‐193aa is highly expressed in glioblastoma, which may be an important biomarker for the formation of glioblastoma. SEMA4B‐211aa and EIF6‐224aa have been confirmed to regulate the formation and development of breast cancer.^16^, ^17^, ^18^ CircRNA regulates the transcriptional extension of multiple oncogenes by encoding protein products, and plays a certain role in promoting or suppressing cancer. CircRNAs' current functions include: (1) regulating gene expression and selective splicing; (2) acting as a sponge for miRNA; (3) interacting with RNA‐binding proteins; (4) translating into proteins or polypeptides. The above biological functions are gradually recognized by most scholars, but of course, there are still biological functions to be explored.^19^, ^20^ It is crucial to emphasize that aberrant circRNA expression is found in practically all cancer types and is essential for the etiology of cancer. Whether as oncogenes or anti‐oncogenes, circRNAs can regulate the proliferation, migration, invasion, and apoptosis of cancer cells through a variety of mechanisms.^21^ For example, hsa_circ_0001020 serves as a potential biomarker for gastric cancer screening and prognosis;^22^ hsa_circ_0030998 promotes tumor proliferation and angiogenesis by sponging miR‐567 to regulate VEGFA in colorectal cancer;^23^ CircDIDO1 suppresses PRDX2 protein stability by encoding a novel DIDO1‐529aa protein and regulates PRDX2 protein stability to inhibit gastric cancer progression;^24^ Circular RNA circ β‐catenin aggravates the malignant phenotype of non‐small‐cell lung cancer via encoding a peptide,^25^ among other studies, confirming the broad biological functions of circRNAs. An increasing number of studies have reported that circRNA is a novel RNA that is more stable and highly conserved in tissues and plasma than other RNAs, such as lncRNA and miRNA, making circRNA an effective novel biomarker for noninvasive diagnosis of malignancies.

In this study, we screened a gene molecule hsa_circ_0000702, which is differentially expressed in GC tissues through the GEO database, and thus we speculated whether the expression of this molecule in the serum of GC patients could be used as a biomarker in GC diagnosis and treatment. Our study verified the circulatory properties of hsa_circ_0000702 by Sanger sequencing, Rnase R assay, gDNA, etc. Subsequently, we constructed a qRT‐PCR method to detect hsa_circ_0000702 and performed a methodological evaluation of the constructed method, and the results showed that the constructed method has high accuracy, precision, stability, and reproducibility. This laid the methodological foundation for our subsequent experiments. Further experiments showed that hsa_circ_0000702 was lowly expressed in GC cell lines (MKN‐45, SGC‐7901, MGC‐803, and AGS), and similarly in serum and tissues of GC patients, while hsa_circ_0000702 expression was higher in gastritis and healthy controls. The expression of hsa_circ_0000702 was higher in gastritis and healthy controls, but the difference between its expression in gastritis and healthy controls was not yet statistically different. This suggests that hsa_circ_0000702 may function as an oncogene in GC. Subsequently, we analyzed the correlation between serum hsa_circ_0000702 and clinicopathological parameters and found that the expression level of serum hsa_circ_0000702 correlated with the degree of tumor differentiation and TNM stage, which revealed that hsa_circ_0000702 was associated with the progression of GC. There are few biomarkers used to diagnose gastric cancer, CEA is one of the common tumor markers, but there is usually no significant change in the early stage of the tumor, and its CEA is usually elevated in the smoking population. In addition, besides tumors, CEA is also elevated in benign diseases such as inflammatory bowel disease, pancreatitis, autoimmune diseases, and other benign diseases. Since CEA is less sensitive in the early stage of GC,^26^ it cannot be used as a biomarker for early confirmation of GC; CA19‐9 is also one of the common tumor markers, produced by adenocarcinoma cells, and is mainly found in tumors such as pancreatic, gastric, and colorectal cancers, but is more significantly elevated in patients with tumors such as pancreatic and biliary tract, and may also be elevated in a variety of benign diseases such as pancreatitis, cholelithiasis, and thyroid.^27^ Therefore, the sensitivity of all these markers is relatively low in early GC.^28^ To further validate the clinical diagnostic efficacy of hsa_circ_0000702 as a potential clinical test, we plotted ROC curves and found that the AUC values of hsa_circ_0000702 were significantly higher than those of common tumor markers for GC (CEA, CA 19–9, etc.), showing that its diagnostic efficacy was superior to that of CEA and CA19‐9. Meanwhile, combined at the same time, we found that the combination of these three diagnostics significantly improved the diagnostic value of GC. Later, we found that the serum expression level of GC patients increased 1 week after surgery compared with the initial diagnosis by dynamic monitoring, which indicated the potential of hsa_circ_0000702 for dynamic monitoring. Next, to explore the possible mechanism of action of hsa_circ_0000702 in GC cells, we first determined the localization of hsa_circ_0000702 in the cell line. hsa_circ_0000702 was mainly localized in the cytoplasm. Numerous studies have shown that circRNAs localized in the cytoplasm usually play the ability to act as miRNA sponges. Subsequently, we further predicted the mechanism of hsa_circ_0000702‐miRNA‐mRNA action. Among these predicted miRNAs, hsa_miR‐708‐5p targets ZNF549 in colon cancer to upregulate and inhibit colon adenocarcinoma proliferation and migration via the PI3K/Akt signaling pathway.^29^ This suggests that the predicted target gene of hsa_circ_0000702, hsa_miR‐708‐5p, may also have a different regulatory network in the development of GC.

According to the clinicopathological characterization of hsa_circ_0000702, hsa_circ_0000702 correlated with the degree of tumor differentiation and TNM stage, which suggests that hsa_circ_0000702 may be involved in the malignant progression of GC cell proliferation, migration, and invasion, giving us a new research direction. Therefore, to fully explore the potential value of hsa_circ_0000702 in GC clinic, further studies should focus on multidistrict collaboration, larger case collection, long‐term follow‐up, and whether hsa_circ_0000702 as ceRNA is involved in regulating GC cell proliferation, metastasis, and invasion. The circRNA‐miRNA‐mRNA regulatory axis predicted by bioinformatics databases also needs further validation to deepen the understanding of the role of hsa_circ_00000702 in GC progression, thus implying the value of hsa_circ_0000702 as a potential therapeutic target for GC.

In conclusion, hsa_circ_0000702 may act as a tumor suppressor gene in GC patients. And in the long run, circRNA may become part of routine clinical workup, especially for some tumors lacking traditional biomarkers, circRNA harbors great clinical value. Of course, in this study, the data we obtained is only a preliminary estimate of the clinical application of hsa_circ_0000702 in GC, and there are inevitably some limitations, such as (1) all cases in this study were only from Nantong University Hospital, and there is a certain chance of specimens; (2) the sample size is relatively small; (3) the lack of long‐term follow‐up; (4) the lack of standardized protocols to confirm whether hsa_circ_0000702 can be applied in a clinical setting.

## AUTHOR CONTRIBUTIONS

WTY, RHF, and HC contributed to the experimental design. WTY, RHF, CYM, HMC, and BJT contributed to clinical diagnosis and sample collection. WTY and RHF contributed to testing. HMC, BJT, and HC contributed to the data analysis, result interpretation, and writing. All authors have reviewed and approved the final study.

## CONFLICT OF INTEREST

The authors report no conflicts of interests in this work.

## DATA AVAIL ABILIT Y STATEMENT

The data used in the current study are available from the corresponding author upon reasonable request.
